# A Case of a Mirror Image Artifact in the Forearm

**DOI:** 10.5334/jbsr.3641

**Published:** 2024-07-05

**Authors:** Miloud Dewilde, Peter Brys

**Affiliations:** 1 UZ Leuven, Leuven, Belgium; 2 UZ Leuven, Leuven, Belgium

**Keywords:** Ultrasound imaging, artifacts, mirror image artifact, reverberation artifact, misinterpretation

## Abstract

*Teaching point:* To emphasize the importance of recognizing mirror image artifacts in musculoskeletal ultrasound to avoid misdiagnosis, unnecessary interventions, and additional diagnostic procedures that can lead to patient anxiety, increased healthcare costs, and potential harm.

## Introduction

The mirror image artifact violates the assumption that the ultrasound waves travel in a linear path and that the echo returns to the transducer after a single reflection [1]. Mirror image artifacts occur when the primary beam of ultrasound waves hits a highly reflective surface, sometimes obliquely oriented, and is reflected by this surface. But on its way, it encounters another structure and is reflected back again toward the highly reflective surface (e.g., bone). It is then reflected again toward the transducer. The transducer considers the delayed echo as reflected from a deeper structure, thus creating the mirror image artifact on the adverse side of the reflecting surface. The transducer software is not able to interpret that the echoes traveled an indirect course on their return path [1].

## Case Report

A 30-year-old man presented to an emergency department 4 days earlier after an injury during a basketball game. His elbow collided with an opponent’s mandible and teeth. An open wound and swelling were visible at the elbow, and the wound was sutured after local disinfection.

Two days later, the patient had a fever and a marked increase in swelling of the elbow with redness. The general practitioner on duty started oral antibiotic therapy.

On the fourth day after the trauma, the patient visited the emergency department because of the steadily increasing swelling and pronounced redness. The lesion was painless at rest but painful on mobilization.

The redness was more extensive, and the mobility of the elbow was markedly reduced. He had a fever of 38.2°C.

An X-ray examination of the elbow revealed no fracture. Adjacent to the ulna, a small air bulb was observed without other discernible abnormalities (Figures 1A and 1B).

**Figure 1A:**
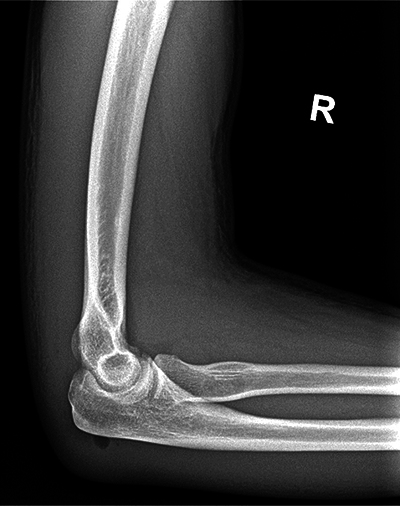
Lateral view of the right elbow.

**Figure 1B:**
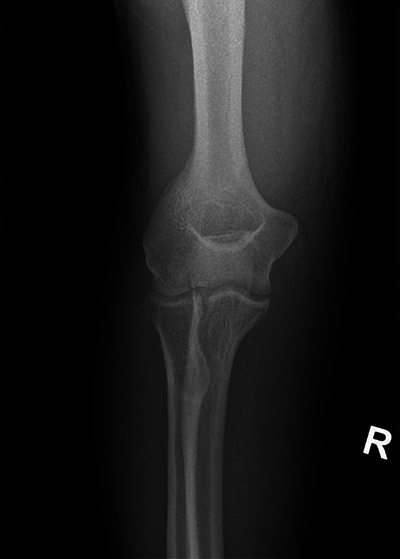
Anterior view of the right elbow.

**Figure 2:**
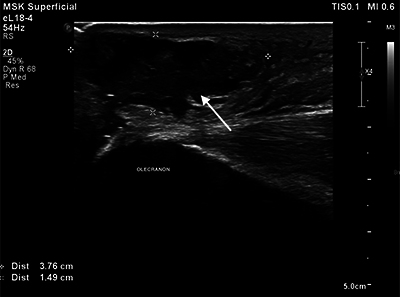
Abscess (arrow) situated adjacent to the olecranon on the ultrasound study.

**Figure 3:**
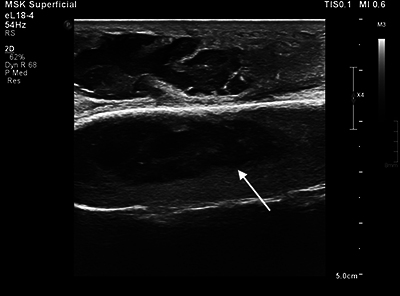
Mirror image artifact of the above-situated true abscess (white arrow), creating the impression of the abscess extending into the olecranon.

An ultrasound examination of the elbow was performed, showing an extensive abscess situated adjacent to the olecranon (Figure 2) with a volume of approximately 13 mL. The content consisted of fluctuating material, most likely pus. Suspicion of the abscess extending into the olecranon was reported (Figure 3).

**Figure 4:**
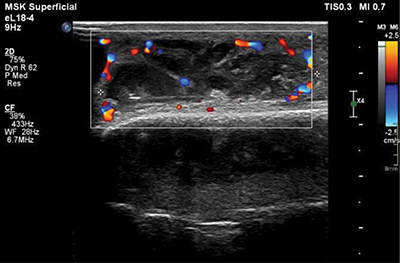
Color Doppler ultrasound examination illustrating the marked hyperemia at the periphery of the collection.

Color Doppler ultrasound revealed marked hyperemia in the entire elbow region (Figure 4), with the most pronounced hypervascularity observed surrounding the abscess. The subcutaneous adipose tissue was markedly hyperechoic and accompanied by diffuse subcutaneous edema, indicative of cellulitis.

Following discussion with a staff radiologist, the diagnosis of an abscess without deep extension was established. It was stressed that the ultrasound image was affected by a mirror artifact, falsely depicting an overlay over the bone of the ulna.

## Discussion

In this case, the ultrasound beam is reflected by the ulna and creates a mirror image artifact of the superficial abscess, simulating extension into the bone.

This is a nice illustration of an infrequent mirror image artifact. There are other more known examples of mirror artifacts, such as the spleen/liver reflecting above the diaphragm in the thoracic space, ascites reflecting over the diaphragm mimicking a pleural effusion, the reflection of the gestational sac mimicking a heterotopic pregnancy, or a duplication of the uterus.[2]

The mirror image artifact may largely mimic the original structure, but it may also appear (partially) weaker. The image of the original structure may also be slightly distorted, or it may also be seen while the primary structure is hidden on that ultrasound image.

If this artifact is not properly recognized, it may be misinterpreted as true pathology. This can largely influence therapeutic management and decisions, with additional unnecessary examinations and interventions. [3]

By changing the angle of the primary ultrasound beam, the artifact may disappear. By alternating the degree of external compression of the transducer on the skin, the content of the abscess fluctuates, simultaneously visible in the false mirror image. This is an argument in favor of an artifact rather than a true extension of the pathology.

## Conclusion

By understanding the principles of mirror image artifacts in ultrasound imaging and with growing experience in ultrasound examinations, artifacts can be differentiated from true pathology, thus improving diagnostic accuracy and patient care and avoiding unnecessary additional examinations and interventions.
